# Cost of diabetes and hypertension care among patients in rural Bangladesh: a cross-sectional study

**DOI:** 10.1186/s12889-026-26456-8

**Published:** 2026-02-04

**Authors:** Baby Naznin, M M Fardeen Kabir, Wubin Xie, Zahidul Quayyum, Tanmoy Sarker, Ali Ahsan, Md. Mokbul Hossain, Aysha Anan, Mst. Jakia Sultana, Lora L. Sabin, Brian Oldenburg, John Chambers, Malay Kanti Mridha

**Affiliations:** 1 BRAC James P Grant School of Public Health, BRAC University, Dhaka, Bangladesh; 2 Nanyang Technological University, Singapore, Singapore; 3 Department of Global Health, School of Public Health, Boston University, Boston, USA; 4 Baker Heart and Diabetes Institute, Melbourne, Australia; 5 School of Psychology and Public Health, La Trobe University, Melbourne, Australia; 6 Department of Epidemiology and Biostatistics, School of Public Health, Imperial College London, London, UK

**Keywords:** Cost of care, Diabetes, Hypertension, Bangladesh, Direct and indirect cost, Financial burden

## Abstract

**Background:**

Hypertension and diabetes impose significant health and economic burdens in low-and middle-income countries like Bangladesh. This study aimed to estimate both direct and indirect costs associated with hypertension and diabetes care in Dinajpur, a rural district of Bangladesh, and identify factors influencing these costs.

**Methods:**

This cross-sectional study used baseline data from a community survey conducted as part of an ongoing implementation research project. A multistage cluster sampling approach was used to randomly select adults aged 40 years and above from 45 wards across three subdistricts of Dinajpur. The analysis included 832 individuals who reported being on medication for hypertension (*n* = 635) and/or diabetes (*n* = 335). Data were collected through structured questionnaires, capturing direct (medical and non-medical) and indirect costs (productivity losses). Descriptive statistics, Wilcoxon rank-sum, and Kruskal-Wallis tests were used for univariate analyses. Multivariate linear regression models with log-transformed cost data were used to identify cost determinants.

**Results:**

The average monthly total cost per patient was BDT 1,308 (USD 10.8) for hypertension and BDT 2,064 (USD 17.1) for diabetes. For both conditions, direct medical costs accounted for around 80% of total costs (60% for medicines), direct non-medical costs around 11% (mostly food and travel), and indirect costs approximately 9%. Direct costs were lower at public facilities compared to private dispensaries (Hypertension: GMR 0.46, 95% CI 0.33–0.64; Diabetes: GMR 0.15, 95% CI 0.10–0.24), while higher costs were observed for private clinics and NGO facilities. Level of education was associated with higher direct costs, particularly among patients with primary or secondary or higher education. Comorbidities were also associated with higher direct costs: in hypertension, cardiovascular disease (GMR 1.78, 95% CI 1.35–2.35) and high cholesterol (GMR 2.16, 95% CI 1.49–3.14) increased direct costs, with similar associations for diabetes costs. Indirect costs, reflecting productivity losses, were higher for private clinics, public facilities, and NGO facilities compared to private dispensaries.

**Conclusions:**

In the sample taken from a rural region from Bangladesh, hypertension and diabetes care entails a considerable financial burden, driven largely by medicine costs and reliance on private healthcare providers. Improved access to essential services and financial protection strategies are needed to reduce out-of-pocket expenditures.

**Supplementary Information:**

The online version contains supplementary material available at 10.1186/s12889-026-26456-8.

## Background

Noncommunicable diseases (NCDs), including hypertension and diabetes, are major global health challenges, accounting for 74% of deaths worldwide in 2023, with 86% of premature deaths occurring in low and middle-income countries (LMICs) [1]. These chronic conditions impose a growing economic burden on individuals and health systems, particularly in LMICs, where healthcare access remains limited [2]. In Bangladesh, the prevalence of diabetes and hypertension among adults (18 + years) is high and increasing, with current estimates of 14% for diabetes and 29% for hypertension [3–5].

Managing diabetes and hypertension in rural Bangladesh is particularly challenging due to limited healthcare access, workforce shortages, and constrained resources [6]. Public facilities, while offering free or low-cost services, are often underfunded and overburdened [7], leading patients to seek care from private providers or unqualified practitioners, which increases household healthcare spending [8, 9]. In Bangladesh, out-of-pocket (OOP) expenditures account for nearly 74% of total health spending, one of the highest proportions globally [10, 11]. Chronic conditions requiring long-term management impose substantial economic burdens through cost of consultations, diagnostics, medications, transportation, and lost income [12], with affected households more likely to face catastrophic health expenditures [13]; for example, 46% of individuals with hypertension experience catastrophic expenditure at the 10% threshold, and one in four report unmet healthcare needs [14]. Multimorbidity, affecting around 30% of adults with hypertension, further increases treatment complexity and household costs [15]. Rural populations, constrained by poverty, limited insurance, and weak social safety nets, are especially vulnerable to these challenges, ultimately resulting in poorer health outcomes [16–18].

While several studies have documented the prevalence, risk factors, and healthcare utilization patterns for diabetes and hypertension in Bangladesh [19, 20], there remains a significant gap in understanding the economic implications of managing these conditions, particularly in rural settings. Afroz et al. [21] found that the average annual cost per person with type 2 diabetes mellitus in Bangladesh in 2017 was USD 864.7, which is about 52% of the country’s per capita GDP in 2017. Hossain et al. [17] estimated the annual OOP cost for diabetes at USD 323, with 75% of costs attributed to medicines posing a significant financial burden on low income households. Much of the existing literature relies on national-level data, which does not capture the rural challenges and indirect costs such as lost income and productivity, which in LMICs can account for 20–50% of the total economic burden [22]. Understanding these costs is crucial for rural Bangladesh, where access to affordable, and quality care is limited. Therefore, this study aimed to assess the direct and indirect costs of seeking care for hypertension and diabetes in Dinajpur, a predominantly rural district of Bangladesh, and identify factors influencing these costs.

## Methods

### Study design and setting

This cross-sectional study used baseline data collected in the Dinajpur district of the Rangpur division, Bangladesh, as part of an ongoing effectiveness-implementation trial to assess the effectiveness and implementation strategy of a digital technology-supported decentralized hypertension and diabetes care model to address gaps in the care continuum [23, 24]. Rangpur is among the poorest Divisions in Bangladesh with 439 subdistricts characterized by predominantly rural populations and limited healthcare resources. The primary healthcare system in these areas is typical of rural Bangladesh, with approximately one NCD corner to 30–40 community clinics. The study was conducted in three subdistricts of Dinajpur district: Biral, Parbatipur, and Chirirbandar (Supplementary Figure 1). These subdistricts were selected to capture diversity in socioeconomic conditions, population growth, and access to health services across rural settings. Biral is primarily agrarian with an estimated population growth rate of 1.07%, Parbatipur is semi-urban with a rate of 1.15%, and Chirirbandar is moderately rural with 0.97% growth [25]. Besides, the BRAC JPG School of Public Health (BRAC JPGSPH), BRAC University has established a strong working relationship with local officials of these subdistricts through collaboration with earlier research programmes, which allows a safe and stable accessibility, and availability of administrative resources for research project management.

### Sampling strategy and sample size

The baseline data included a sample of 6849 adults aged 40 years and above who were randomly selected from 45 wards across the three selected subdistricts. A three-stage sampling design was employed. In the first stage, 45 wards (15 from each subdistrict) were selected using stratified random sampling proportional to union population size. In the second stage, each selected ward was divided into segments of approximately 250 households, from which one segment was randomly selected. In the third stage, a household listing was prepared to identify all male and female adults aged 40 years and above, and a simple random sample of 88 men and 88 women was drawn from each subdistrict, accounting for an expected 15% non-response rate.

### Study participants and eligibility criteria

Adults aged 40 years and above who were permanent residents (living ≥6 months) of the selected wards and had a self-reported physician diagnosis of hypertension and/or diabetes were included. Hypertension was defined as if a participant had systolic blood pressure 140 mmHg or more, or diastolic pressure 90 mmHg or more, or both and/or history of hypertension and/or currently on medication for hypertension [26]. Diabetes was defined as having fasting blood glucose level ≥ 7 mmol/L, ever received a diagnosis of diabetes, or currently on medication for diabetes [23]. 

Participants were excluded if they were critically ill at the time of data collection, pregnant, unable to communicate due to severe physical or cognitive impairments or unwilling to provide informed consent. For this particular study, we restricted analyses to participants who, during the survey, reported being on medication for hypertension and/or diabetes. The final analytic sample thus consisted of 832 participants, with 635 individuals receiving treatment/medication for hypertension and 335 individuals with diabetes (including 138 participants who had both hypertension and diabetes; Supplementary Figure – 2).

### Data collection and measurements

#### Data collection tool

The questionnaire was adapted from economic evaluation tools previously used by our research team over the past 15 years. It was designed to capture standard components of direct costs (registration, consultation, diagnostics, and medications) as well as other expenses incurred when seeking care (e.g., transportation, food, and informal payments). The tool also incorporated indirect costs, including lost time and productivity for both patients and any accompanying attendants. All cost information was collected using a 30-day recall period. In addition to cost data, the questionnaire gathered information on participants’ sociodemographic characteristics, medical history, and care-seeking behaviors related to hypertension and diabetes. For this study, the tool was refined to align with the research objectives, translated and pilot-tested in the local language, and further adjusted based on pilot findings. Although not formally validated, the tool’s comprehensive coverage of key cost items reflects standard methodology used in multiple peer-reviewed studies [27, 28]. 

#### Data collection procedures

Data was collected between January and March 2024 through structured household surveys administered by trained enumerators. Twelve enumerators underwent a five-day intensive training program conducted by senior researchers from BRAC JPGSPH. The training covered research ethics, interviewing techniques, detailed questionnaire administration, and cost data collection procedures. The training also included instructions on conducting anthropometric measurements, blood pressure assessments, and fasting blood glucose tests. Blood glucose and blood pressure measurement

#### Blood glucose and blood pressure measurement

Blood glucose data were collected using Accu-Chek Active^®^ digital glucometers following standardized finger-prick protocols. Quality control measures included daily calibration of each device with control solutions, verification of 10% of samples by field supervisors, replacement of any glucometer that showed > 10% deviation in repeated readings, and daily data cross-checking of readings with participant IDs. Blood pressure was measured following standardized protocols. Three readings were taken on the right arm at 1-minute intervals, and all measurements were recorded. Participants with blood pressure readings > 180/110 mmHg were referred for urgent medical review.

### Outcome variables

The outcome variable was the total cost incurred by individuals seeking care for diabetes and hypertension, consisting of both direct and indirect costs. Direct costs were defined as all out-of-pocket expenditures borne by patients, including medical expenses (registration, consultation, diagnostic tests, and medications) and non-medical expenses (transportation, food, and informal payments). Indirect costs captured productivity losses and were defined as the value of time lost by patients and any accompanying attendants due to travel, waiting, and receiving care [29]. Together, these direct and indirect components constituted the total cost of care.

#### Measurement of costs

Direct costs were calculated by summing all medical and non-medical expenses reported by participants for each healthcare visit within the past 30 days. Indirect costs were measured using the human capital approach [30, 31], that values productivity losses based on the opportunity cost of time. Opportunity cost was defined following Palmer and Raftery [32] as the value of the alternative activity forgone due to seeking care. Indirect cost calculation followed the steps below:

*Measurement of lost time*: During data collection, enumerators recorded the time spent (in minutes, converted in hours where needed) by the patient (and any accompanying attendants) on travel (round trip), waiting, and consultation/diagnostic procedures for each health facility visit.

*Total time lost*: For each participant, total time lost per visit was calculated by summing hours spent on travel, waiting, and consultation. When an attendant was present, the attendant’s time was added to the patient’s total time. Thus, total lost time = patient time (hours) + attendant time (hours).

*Valuation of time*: Value of lost time was calculated using the national monthly minimum wage [33] as a conservative proxy for the opportunity cost of time. The hourly wage was derived by dividing the monthly minimum wage by 26 working days and 8 working hours per day (i.e., 208 h/month).

*Calculation of indirect costs*: Total indirect cost was calculated as: (Total hours lost by patient + total hours lost by attendant) × hourly wage. For participants reporting multiple visits during the recall period, indirect costs were summed across all visits to obtain the total indirect cost per participant.

### Explanatory variables

The explanatory variables included a range of sociodemographic factors, comorbidities, and sources of healthcare, all of which have been shown in previous studies to influence both the prevalence and economic burden of chronic diseases [34]. Sociodemographic factors included age (40–49, 50–59, 60 years and older), gender (male, female), religion (Muslim, non-Muslim), marital status (currently married, single/widowed/divorced/separated), level of education (no formal schooling, primary, secondary or higher), and employment status (self-employed/homemaker, employed/retired, unemployed). Socioeconomic status (SES) was assessed via a wealth score derived from principal component analysis of household assets, divided into low, medium, and high categories. Comorbidities included self-reported conditions (ever diagnosed by a healthcare professional) including cardiovascular disease (CVD), chronic respiratory disease (CRD), and high cholesterol. Sources of healthcare were categorized as public facilities, NGO facilities, private clinics, and private dispensaries. Public facilities included government hospitals and health centers, whereas NGO facilities referred to services provided by non-governmental organizations. Private clinics were defined as formal for-profit healthcare facilities owned and operated by individuals, groups, or organizations offering medical consultations, diagnostic services, and treatment, and are staffed by medical practitioners with formal training. Private dispensaries, on the other hand, are smaller facilities, such as local pharmacies or drug shops, that mainly provide medications and informal health advice, often without full-time doctors.

### Statistical analysis

Descriptive statistics, presented as frequencies (n) and percentages (%), were used to summarize the characteristics of the study participants. Mean and standard deviation (SD) were calculated for both direct and indirect costs and reported in Bangladeshi Taka (BDT). For comparisons across categorical variables, the Wilcoxon rank-sum (Mann–Whitney) test was used for comparing two groups, whereas the Kruskal–Wallis test was applied for comparisons involving more than two groups. Univariate analysis was conducted to explore associations between cost variables and explanatory factors, such as age group, gender, education, employment status, SES, source of care, and comorbidities.

A multivariate linear regression model was used to examine the factors influencing the total cost of care for patients with hypertension and diabetes. Natural logarithm transformation of the outcome variables was performed as the outcome measures were positively skewed. Regression analyses were conducted separately for the direct and indirect costs. As log-transformation was used, the geometric mean ratios (GMR) were derived by exponentiating the coefficients. Statistical significance was assessed at the 5% level (*p* < 0.05), with robust standard errors adjusted for clustering; 95% confidence intervals were reported. Zero-cost cases (< 5% of data) were excluded from the main regression analyses, as log transformation is undefined for zero values. To determine the robustness of our findings to the exclusion of zero-cost cases, a sensitivity analysis was conducted by adding a small constant (1 BDT) to include these observations in the log-transformed regression models. In a secondary analysis, we estimated average costs for three mutually exclusive patient groups, namely those with hypertension only, those with diabetes only, and those with both conditions. All data were analyzed using Stata/MP 17.0.

## Results

### Characteristics of the participants

Table 1 presents the demographic and clinical characteristics of the participants with hypertension (*n* = 635) and diabetes (*n* = 335). A large proportion of participants with both conditions were aged 60 years and above, with 49% of hypertensive patients and approximately 41% of diabetic patients in this age group. The gender distribution was skewed towards females, with women representing 55.4% of patients with hypertension and 53.7% of patients with diabetes. The majority of hypertension (81.7%) and diabetic (87.8%) patients were married. Almost half of hypertensive patients (49.0%) and diabetic patients (48.1%) had completed primary school. Most of the respondents in both groups were self-employed or homemakers (82.4%). CVD was reported in 14.8% of patients with hypertension and 7.8% of patients with diabetes. Private dispensaries/pharmacies were the most common source of care for hypertensive patients (42.7%), followed by private clinics (34.5%), whereas NGO facilities were more frequently used by diabetic patients (30.8%), followed by private dispensaries and clinics.

**Table 1:** Distribution of sociodemographic & clinical characteristics among patients with hypertension and diabetes

Variables^‡^	Hypertension ^§^ [*n* = 635] [*n*, %]	Diabetes ^§^ [*n* = 335] [*n*, %]
**Demographic**		
** Age group**		
40–49	107 (16.9)	81 (24.2)
50–59	217 (34.2)	117 (34.9)
60+	311 (49.0)	137 (40.9)
** Gender**		
Male	283 (44.6)	155 (46.3)
Female	352 (55.4)	180 (53.7)
** Currently married**		
Yes	519 (81.7)	294 (87.8)
No	116 (18.3)	41 (12.2)
** Religion**		
Muslim	551 (86.8)	297 (88.7)
Non-Muslim	84 (13.2)	38 (11.3)
** Education**		
No formal schooling	177 (27.9)	88 (26.3)
Primary	311 (49.0)	161 (48.1)
Secondary or higher	147 (23.2)	86 (25.7)
** Employment**		
Self-employed or homemaker	523 (82.4)	276 (82.4)
Employed or retired	46 (7.2)	31 (9.3)
Unemployed	66 (10.4)	28 (8.4)
** Socioeconomic status**		
Low	132 (20.8)	51 (15.2)
Medium	160 (25.2)	84 (25.1)
High	343 (54.0)	200 (59.7)
**Clinical characteristics**		
** Disease History/Co-morbidities**		
Cardiovascular disease	94 (14.8)	26 (7.8)
Chronic respiratory disease	52 (8.2)	26 (7.8)
High cholesterol	64 (10.1)	41 (12.2)
** Sources of care**		
Public facility	92 (14.5)	58 (17.3)
NGO facility	53 (8.4)	103 (30.8)
Private clinic	219 (34.5)	85 (25.4)
Private dispensary/pharmacies	271 (42.7)	89 (26.6)

^**‡**^ n and column % were reported. Socioeconomic status was assessed by a wealth score derived from principal component analysis of a set of household assets. Cardiovascular disease, chronic respiratory disease, and high cholesterol were all self-reported chronic conditions ever diagnosed by a healthcare professional

^§^ Hypertension was defined as if a participant had systolic blood pressure ≥ 140 mmHg, or diastolic pressure ≥ 90 mmHg, or having a history of hypertension and currently on medication for hypertension. Diabetes was defined as having fasting blood glucose level ≥ 7 mmol/L, ever received a diagnosis of diabetes, or currently on medication for diabetes

#### Direct and indirect costs of hypertension and diabetes care

Table 2 provides findings on average monthly total cost of care for patients with hypertension and diabetes, disaggregated into direct medical, direct nonmedical, and indirect costs. For hypertensive patients, the average monthly cost was BDT 1,305 (USD 11), while for diabetic patients it was BDT 2,043 (USD 17). Medicines accounted for the largest share of expenditure in both conditions, representing 63% of hypertension costs (BDT 817) and 61% of diabetes costs (BDT 1,237). Diagnostic costs were the second-largest component, contributing 14% for hypertension (BDT 177) and 16% for diabetes (BDT 321), whereas consultation fees were relatively modest (BDT 59 for hypertension; BDT 63 for diabetes). The total direct medical costs amounted to BDT 1,062 (81%) for hypertension and BDT 1,644 (80%) for diabetes.

**Fig. 1:**
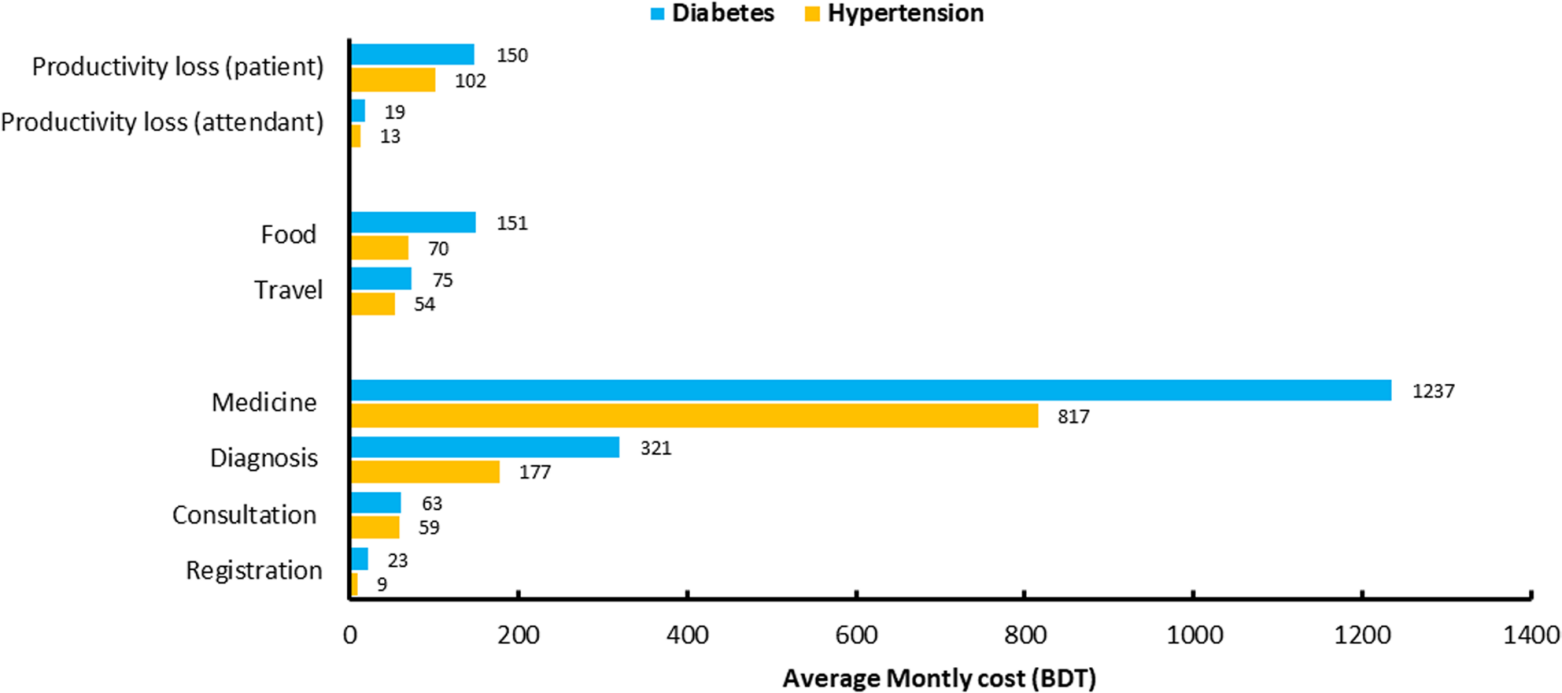
Average monthly direct and indirect costs of hypertension and diabetes care (BDT)

Direct non-medical costs, which mainly include expenses for food and transportation, accounted for 10% of total hypertension costs and 11% of total diabetes costs, with slightly higher spending among diabetic patients. Indirect costs, reflecting lost productivity, were relatively modest but consistently higher for diabetic patients. On average, diabetic patients lost 94 min per month, equivalent to BDT 150, while hypertensive patients lost 64 min per month, equivalent to BDT 102. Direct and indirect costs for both conditions are illustrated in Fig. 1.

In addition, the costs associated with time spent by patients’ attendants were comparatively small (BDT 19 for diabetes and BDT 13 for hypertension). A detailed breakdown of lost time and productivity is provided in Supplementary Fig. 3.

**Table 2:** Monthly direct and indirect costs for hypertension and diabetic care [in BDT]

Cost Items ^‡^	Hypertension ^§ (^*n* = 635)		Diabetes ^§ (^*n* = 335)	
	Mean BDT (95% CI)	% of total cost	Mean BDT (95% CI)	% of total cost
Direct Medical Cost Items				
Registration	9 (2.4–15.1)	0.7%	23 (10.1–35.7)	1.1%
Consultation	59 (43.6–74.5)	4.5%	63.0 (37.6–88.5)	3.1%
Diagnosis	177 (92.2–260.8)	13.6%	321 (153.2–488.2)	15.7%
Medicine	817 (720.7–912.8)	62.6%	1,237 (1007.7–1465.8)	60.6%
Total direct medical cost	**1**,**062 (903.0–1225.4)**	**81.4%**	**1**,**644 (1267.9–2060.7)**	**80.5%**
Direct Non-medical Cost Items				
Travel	54 (37.8–70.0)	4.1%	75 (51.4–99.3)	3.7%
Food cost	70(48.3–92.6)	5.4%	151 (97.4–204.4)	7.4%
Informal costs (gifts, tips)	4 (0.7–7.5)	0.3%	4(0.4–9.1)	0.2%
Total direct non-medical medical cost	**128 (96.3–161.5)**	**9.8%**	**230 (159.4–302.7)**	**11.3%**
Indirect Costs Items				
Cost of lost time/productivity (patient)	102 (88.4–115.1)	7.8%	150 (124.2–175.0)	7.3%
Cost of lost time/productivity (attendant)	13 (7.6–18.0)	1.0%	19.0 (10.3–27.8)	0.9%
Total indirect cost	**115(97.8–131.4)**	**8.8%**	**169 (137.9–199.5)**	**8.3%**
Total cost in BDT	**1**,**305 (1115.9–1499.4)**	**100%**	**2**,**043 (1608.6–2519.4)**	**100%**
Total cost in USD	**11 (9.2–12.4)**		**17 (13.3–20.8)**	

^**‡**^ Arithmetic means with 95% CI and for each cost categories, proportions within total cost reported. Direct costs were defined as all out-of-pocket expenditures borne by patients, including medical expenses (registration, consultation, diagnostic tests, and medications) and non-medical expenses (transportation, food, and informal payments). Indirect costs were defined as the value of time lost by patients and any accompanying attendants due to travel, waiting, and receiving care, representing productivity losses. Indirect costs were measured using the human capital approach to quantify potential productivity losses from seeking care for hypertension and diabetes at different health facilities

^§^ Hypertension was defined as if a participant had systolic blood pressure ≥ 140 mmHg, or diastolic pressure ≥ 90 mmHg, or having a history of hypertension and currently on medication for hypertension. Diabetes was defined as having fasting blood glucose level ≥ 7 mmol/L, ever received a diagnosis of diabetes, or currently on medication for diabetes

### Correlates of the cost of hypertension and diabetes care

Table 3 shows the estimated average total costs (direct and indirect) for hypertension and diabetes across population subgroups defined by sociodemographic characteristics and comorbidities. Regarding direct costs for hypertension care, several factors were associated with higher expenditure: male relative to female (BDT 1,495 versus 950), currently married versus not married (BDT 1,254 versus BDT 919), higher educational attainment (BDT 1,903 for those with secondary education or higher compared with 638 BDT for patients with no formal schooling), higher SES (BDT 1,485 for the top tertile compared with BDT 752 for the bottom tertile), being employed relative to homemaker/self-employed (BDT 2,539 versus BDT 1,048), having CVD (BDT 1,820 versus BDT 1,084) and high cholesterol (BDT 2,637 versus BDT 1,032), and receiving care from non-public facilities (e.g., BDT 1,624 for private clinics versus 602 for public health facilities). Regarding the indirect costs of hypertension care, only education and source of care were significantly associated with self-reported expenditure. Those with secondary or higher education (BDT 135) appeared to have higher indirect cost for hypertension compared with those with no schooling (BDT 77). Private dispensaries appeared to have relatively lower indirect costs (BDT 73).

Turning to the costs of diabetes care, similar patterns were observed for associated factors. For example, those with secondary or higher education had an average cost of BDT 3,357 compared with BDT 896 for patients with no formal schooling; patients who were employed or retired had higher direct costs compared to self-employed individuals or homemakers (BDT 3,115 vs. BDT 1,738). As for the indirect cost of diabetes care, comorbid CVD and source of care were the only significant factors.

**Table 3:** Mean direct and indirect costs (in BDT) for hypertension and diabetes care by socio-demographic and clinical characteristics

Characteristics ^‡^	Hypertension ^§^ (*n* = 635)		Diabetes ^§^ (*n* = 335)	
	Direct cost	Indirect cost	Direct cost	Indirect cost
	Mean (SD)			
Demographic				
Age group				
40–49	1094 (1462)	123 (202)	1874 (5776)	203 (330)
50–59	1132 (2011)	120 (220)	1643 (2499)	139 (194)
60+	1270 (2781)	108 (216)	2123 (3925)	173 (321)
* p-value*	*0.788*	*0.766*	*0.334*	*0.703*
Gender of the participants				
Male	1495 (2339)	133 (259)	2130 (2955)	189 (312)
Female	950 (2335)	100 (170)	1693 (4811)	150 (262)
* p-value*	0.000*	*0.137*	0.001*	*0.095*
Currently married				
Yes	1254 (2467)	117 (220)	1990 (4271)	172 (293)
No	919 (1715)	105 (192)	1215 (1852)	147 (236)
* p-value*	0.002**	*0.404*	0.012*	*0.497*
Religion				
Muslim	1210 (2373)	115 (208)	1945 (4250)	173 (294)
Non-Muslim	1081 (2205)	110 (256)	1509 (2006)	139 (216)
* p-value*	*0.461*	*0.097*	*0.562*	*0.641*
Education				
No formal schooling	638 (1046)	77 (128)	896 (1300)	116 (178)
Primary	1174 (1788)	126 (228)	1661 (2060)	168 (285)
Secondary or higher	1903 (3872)	135 (261)	3357 (7192)	223 (361)
* p-value*	0.000*	0.024*	0.000*	*0.140*
Employment				
Self-employed or homemaker	1048 (2157)	111 (216)	1738 (4181)	164 (291)
Employed or retired	2539 (3876)	146 (211)	3115 (3941)	167 (243)
Unemployed	1405 (2149)	118 (212)	2096 (2548)	217 (287)
* p-value*	0.000*	*0.308*	0.010*	*0.307*
Socioeconomic Status (SES)				
Low	752 (1136)	115 (170)	971 (1195)	180 (243)
Medium	932 (1457)	107 (170)	2118 (6092)	182 (315)
High	1485 (2927)	118 (247)	2037 (3399)	160 (285)
* p-value*	0.000*	*0.478*	0.001*	*0.764*
Personal disease history/Co-morbidities				
CVD				
Yes	1820 (2120)	128 (201)	2984. (2600)	276 (287)
No	1084 (2373)	112 (217)	1804 (4148)	160 (285)
* p-value*	0.000*	*0.657*	0.001*	0.005*
CRD				
Yes	1511 (1972)	118 (202)	2572 (2581)	103 (114)
No	1165 (2380)	114 (216)	1839 (4157)	174 (295)
* p-value*	*0.106*	*0.759*	0.037*	*0.686*
High cholesterol				
Yes	2637 (4979)	191 (301)	3223 (5657)	231 (327)
No	1032 (1773)	106 (202)	1710 (3759)	160 (279)
* p-value*	0.000*	*0.251*	0.001*	*0.271*
Sources of Care				
Public facility	602 (1099)	138 (195)	512 (1013)	174 (214)
NGO facility	1552 (1631)	162 (233)	1556 (1556)	180 (250)
Private clinic	1624 (3490)	144 (273)	3227 (7185)	232 (418)
Private dispensary	976 (1370)	73 (149)	1918 (2584)	92 (178)
* p-value*	0.000*	0.033*	0.000*	0.023*

^**‡**^ Differences in mean costs among subgroups were assessed using the Wilcoxon rank-sum test for binary variables and the Kruskal–Wallis test for variables with more than two categories. Statistically significant differences at *p* < 0.05 are marked with an asterisk. Socioeconomic status was assessed by a wealth score derived from principal component analysis of a set of household assets. Cardiovascular disease, chronic respiratory disease, and high cholesterol were all self-reported chronic conditions ever diagnosed by a healthcare professional

^§^ Hypertension was defined as if a participant had systolic blood pressure ≥ 140 mmHg, or diastolic pressure ≥ 90 mmHg, or having a history of hypertension and currently on medication for hypertension. Diabetes was defined as having fasting blood glucose level ≥ 7 mmol/L, ever received a diagnosis of diabetes, or currently on medication for diabetes

#### Factors associated with the costs of hypertension and diabetes care

**Fig. 2:**
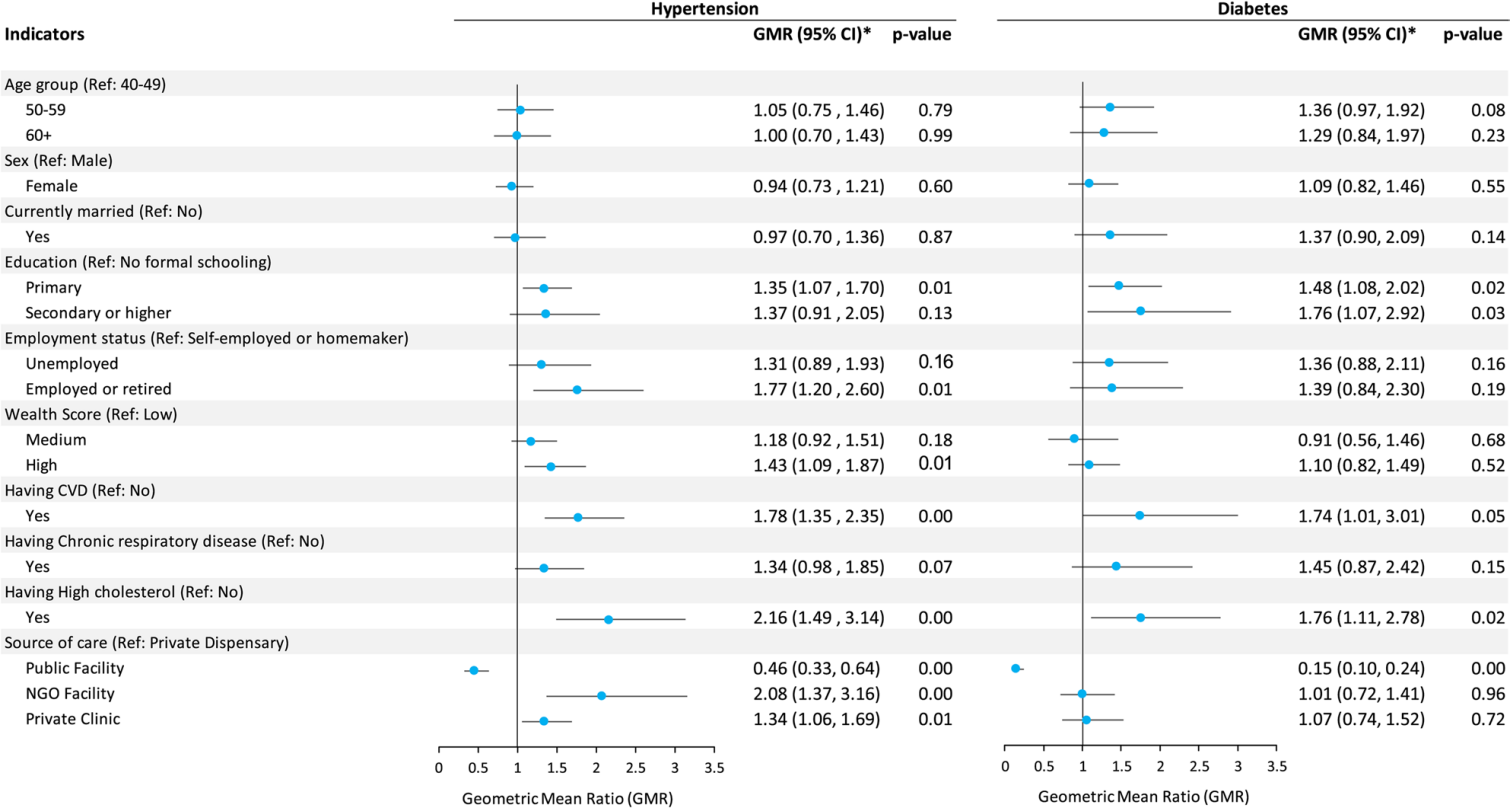
Factors associated with the direct cost of seeking hypertension and diabetes care. * Geometric mean ratios were obtained from exponentiating the beta coefficients from multivariate linear regression models after logarithm transformation of the dependent variable (i.e., direct cost)

**Fig. 3:**
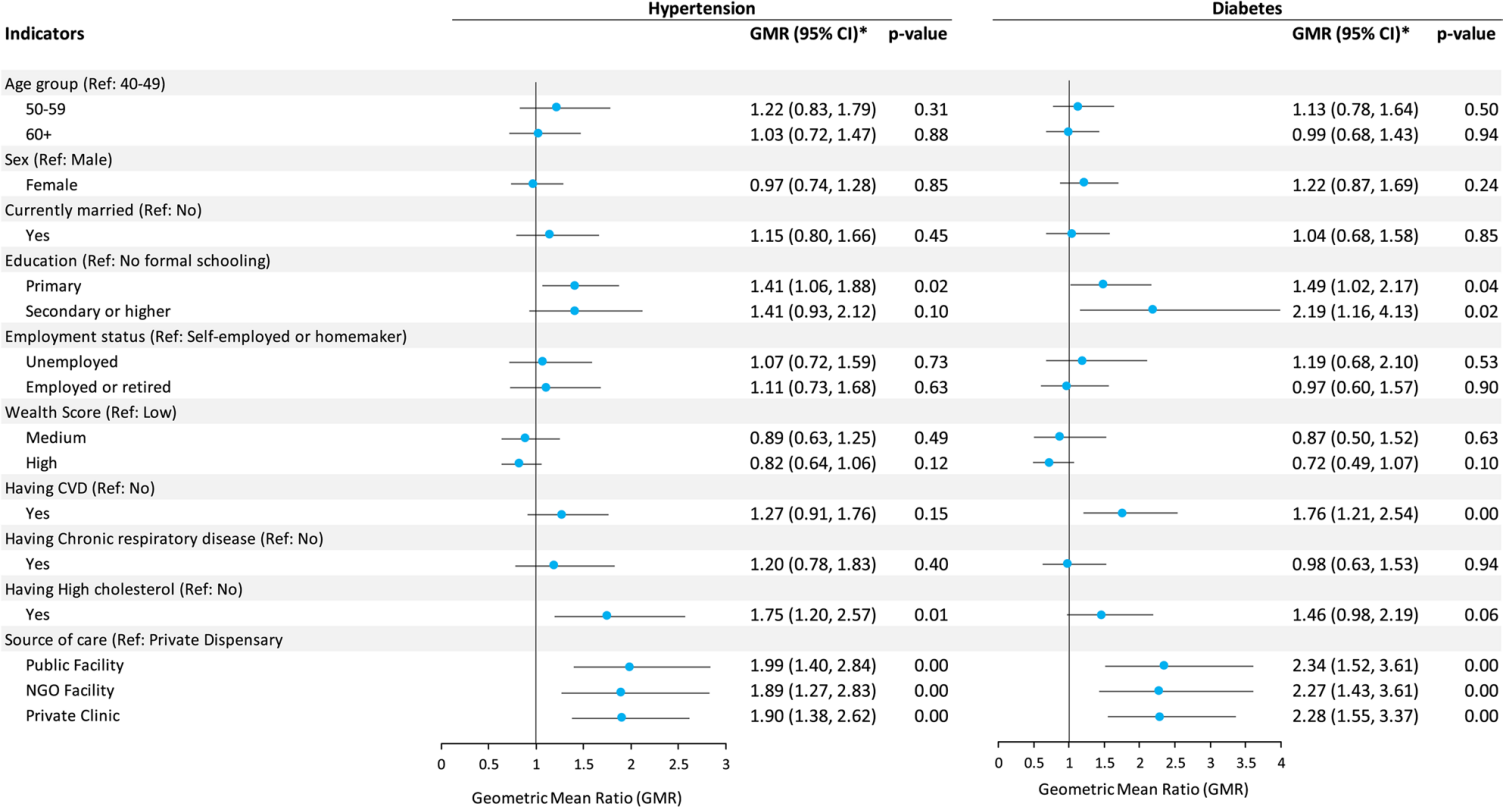
Factors associated with the indirect cost of seeking hypertension and diabetes care. *Geometric mean ratios were obtained from exponentiating the beta coefficients from multivariate linear regression models after logarithm transformation of the dependent variable (i.e., indirect cost).

Figures 2 and 3 present the multivariable-adjusted regression results, illustrating the associations between sociodemographic, clinical, and care setting related factors with the direct and indirect costs of hypertension and diabetes care. The regression coefficients were geometric mean ratios (GMRs), representing the relative differences in geometric mean cost of one category compared with the reference category. Values greater than 1 indicate higher costs, whereas values less than 1 indicate lower costs; error bars represent 95% confidence intervals. Percent changes in costs were calculated from the GMRs as (GMR − 1) ×100, providing a more interpretable measure of cost differences.

Factors significantly associated with direct costs for hypertensive patients included receiving primary education, employment status, SES, having comorbidity (CVD or high cholesterol), and source of care (Fig. 2). Compared with homemakers/self-employed patients, those currently employed or retired had on average 77% higher direct costs (GMR 1.77; 95% CI 1.20–2.60), controlling other factors included in the model. Having CVD (GMR 1.78; 95% CI 1.35–2.35) and high cholesterol (GMR 2.16; 95% CI 1.49–3.14) was associated with markedly higher direct costs. Compared to receiving care from private dispensaries, the average direct costs for hypertension were 54% lower for public facilities, 108% (GMR 2.08; 95% CI 1.37–3.16) and 34% (GMR 1.34; 95% CI 1.06–1.69) higher for NGO facilities and private clinics. Factors associated with direct diabetes cost included education, having comorbid CVD and high cholesterol, and source of care. Adjusted for all factors included in the model, diabetic patients with secondary or higher education had 76% higher cost compared with those who had no formal education (GMR 1.76; 95% CI 1.07–2.92). Having comorbid CVD and high cholesterol were associated with 74% (GMR 1.74; 95% CI 1.01–3.01) and 76% (GMR 1.76; 95% CI 1.11–2.78) higher cost, respectively. Compared with receiving care from private dispensaries, public health facilities had 85% (GMR 0.15; 95% CI 0.10–0.24) lower cost, on average.

Figure 3 presents the factors associated with the indirect costs of hypertension and diabetes care. Factors associated with indirect costs for hypertension care included education, having comorbid high cholesterol, and source of care. The hypertensive patients with primary school education had 41% (GMR 1.41; 95% CI 1.06–1.88) higher cost compared with those with no formal education; patients with comorbid high cholesterol had 75% (GMR 1.75; 95% CI 1.20–2.57) high cost compared with those without; relative to those who seeking care from private dispensary, patients receiving care from public health facilities, NGO facilities, and private clinics had 99% (GMR 1.99; 95% CI 1.40–2.84), 89% (GMR 1.89; 95% CI 1.27–2.83) and 90% (GMR 1.90; 95% CI 1.38–2.62) higher cost, respectively.

Factors associated with the indirect cost of diabetes care (Fig. 3) included education, having comorbid CVD, and source of care. Patients with secondary or higher education had more than double the indirect cost (GMR 2.19; 95% CI 1.16–4.13) of those with no formal schooling. Patients with comorbid CVD had 76% (GMR 1.76; 95% CI 1.21–2.54) higher cost than those with no CVD. Relative to private dispensary, indirect costs were 134% (GMR 2.34; 95% CI 1.52–3.61), 127% (GMR 2.27; 95% CI 1.43–3.61), 128% (GMR 2.28; 95% CI 1.55–3.37) higher for public health facilities, NGO facilities, and private clinics, respectively.

#### Sensitivity and secondary analysis

Sensitivity analyses adding a small value to zero cost yielded largely the same patterns of association (Supplementary Tables 1 and 2). Adding a small value to zero cost in general reduced the precision of the estimations (Model II) compared to the original results (Model I), resulted in several coefficients losing statistical significance. In the secondary analysis categorizing participants into three mutually exclusive groups (Supplementary Table 3), the average total costs appeared to be higher among patients living with both hypertension and diabetes (BDT 2,596) compared with those with either diabetes (BDT 1,691) or hypertension (BDT 950) only.

## Discussion

This study is among the first to report the individual-level cost of hypertension and diabetes care in rural Bangladesh. In the sample taken from a rural region from Bangladesh, patients incurred considerable healthcare expenditure, with average monthly costs of BDT 1,305 for hypertension and BDT 2,043 for diabetes. These expenses represent approximately 5–8% of the median monthly income of rural households [35]. Direct medical costs accounted for the largest share of total costs, followed by direct non-medical and indirect costs. Several socioeconomic and health-related factors, including education, employment, socioeconomic status, comorbidities, and source of care, were found to be associated with higher expenditures.

Our results showed a relatively strong socioeconomic gradient in hypertension and diabetes costs. Higher levels of education and socioeconomic status were associated with higher costs for both conditions. This likely reflects greater ability to pay, greater awareness of comorbidities, and higher utilization of private-sector providers, including specialist services. Conversely, individuals from lower socioeconomic status may delay care-seeking or opt for low-cost providers, reducing expenses but potentially compromising health outcomes. In a social context where health insurance and universal health care do not exist, the access (or lack thereof) to primary care and trained professionals may affect socioeconomic groups differently, leading to exacerbated health inequality.

Comorbidities such as CVD and high cholesterol were significantly associated with higher costs for both hypertension and diabetes. This is expected given the need for more frequent monitoring, additional medications, and potential complications increasing costs of care. The substantially higher costs observed among individuals living with both hypertension and diabetes further highlight the dual burden faced by patients with multimorbidity. This finding aligns with the growing literature showing that multimorbidity markedly increases financial burden and the risk of catastrophic health expenditure in LMICs [36]. Source of care was another significant factor influencing the costs of hypertension and diabetes. Patients receiving services from private clinics or NGO facilities incurred higher direct costs (two to six times) compared with those using public facilities, which provide consultations and medicines at free or subsidized cost [17]. Despite the higher financial burden associated with private providers, most patients in our study relied on private dispensaries and clinics, underscoring the limited availability, accessibility, or perceived quality of services in the public sector [19].

Indirect costs, although smaller (8–9% of total costs), remain a significant burden. Our findings highlight the substantial time burden and lost income involved in care seeking. Patients with chronic conditions need frequent follow-up with healthcare providers, leading to loss of productivity. We found that managing hypertension or diabetes in the presence of comorbidity (e.g., CVD, high cholesterol) may require more time and resources, contributing to higher indirect costs. Our results indicated that seeking care from private dispensary was associated with the lowest indirect costs. This is expected because of the geographical proximity and shorter travel time required to access care at such facilities.

Our findings are broadly consistent with a few previous studies conducted in Bangladesh. For example, using a national sample from the Bangladesh Household Income and Expenditure Surveys (HIES), Khan et al. [37] reported monthly out-of-pocket direct costs of BDT 2,160 for diabetes and BDT 3,601 for hypertension, slightly higher than observed in the present study. Previous studies also showed that cost of medication consistently accounted for the largest proportion within total healthcare expenses. Afroz et al. [21] reported that medicine costs comprised more than 60% of diabetes-related expenditures, while Hossain et al. [17] found an even higher proportion (75%). These results align with the present study, where medications accounted for approximately 60% of direct medical costs for both diabetes and hypertension. Diagnostic costs emerged as the second-largest cost driver in our study, about 16% for diabetes and 14% for hypertension, reflecting routine monitoring and screening for complications. In contrast, studies from Kenya and other LMICs [38–40] found transportation as the second-largest contributor to total cost, likely due to longer travel distances. The relatively modest travel costs observed in our study (around 4–5%) may be partly explained by shorter distances and lower transport costs.

The findings of this study underscore several implications for public health policy and service delivery within rural settings like Dinajpur in Bangladesh. First, the high reliance on private dispensaries and clinics, driven by overcrowding, limited availability of medicines, and short operating hours in public facilities, suggests an urgent need to strengthen public-sector capacity. Ensuring consistent availability of essential medicines for hypertension and diabetes and improving service readiness at public facilities could substantially reduce OOP expenditures in Dinajpur and similar contexts. Second, the time and productivity loss associated with care seeking highlights the importance of decentralizing NCD services. Community-based screening, task-sharing with community health workers, and digital health tools for follow-up could reduce indirect costs and improve adherence, particularly for patients with multimorbidity. Third, given that a significant portion of spending occurs in the private sector in our study area, stronger regulation, integration of private providers into referral networks, and adoption of standardized clinical protocols are needed to improve quality of care and prevent unnecessary expenditures.

This study had several limitations. First, the data on health service utilization, treatment costs, and productivity losses were self-reported by the participants. Thus, recall and social desirability bias were possible, which is common in survey-based studies. Second, while the questionnaire was adapted from standard cost of care and analysis tools and pilot-tested in our study context, it was not formally validated which may affect our cost estimates and limit the comparability of our findings with studies employed different data collection instruments. Third, the cross-sectional nature of the study limited causal inference between the costs of care and their associated factors. While a relatively comprehensive list of covariates was included in the regression analyses, unmeasured and uncontrolled confounding factors cannot be ruled out. One of these may be geographical distance to healthcare facilities, as it could influence care seeking behavior and associated costs, although travel time was included as part of indirect cost estimation. Fourth, due to data limitations, we were unable to assess the variation in the costs of care based on hypertension and diabetes severity, although higher costs associated with multimorbidity is indicative of a likely positive relationship between disease severity and costs. Finally, the study was conducted in a specific rural area (Dinajpur) of Bangladesh, which may limit the generalizability of the findings to other regions (e.g., urban settings or rural areas with different healthcare delivery systems) or populations with different socio-economic contexts, or disease prevalence patterns.

## Conclusion

The present study indicated that managing hypertension and diabetes in rural settings like Dinajpur, Bangladesh, imposes a substantial financial burden on patients, with costs varying by sociodemographic characteristics, comorbidities, and sources of care. Direct medical expenses, particularly for medicines, constitute the largest share of out-of-pocket costs, while indirect costs such as travel, waiting time, and productivity loss add a significant, often overlooked, dimension to the economic burden. Diabetes care is notably more expensive than hypertension, likely reflecting the complexity of treatment and care-seeking patterns. Strengthening the public healthcare system, improving access to affordable medicine, and addressing both direct and indirect costs are critical to mitigate financial burdens for patients and to address gaps in care continuum.

## Supplementary Information

Supplementary Material 1.

## Data Availability

The datasets used and/or analyzed during the current study are available from the corresponding author on reasonable request.

## Abbreviations

NCDs: Non-Communicable Diseases
LMIC: Low- and Middle-Income Country
OOP: Out of Pocket
CVD: Cardiovascular Diseases
CRD: Chronic respiratory disease
SES: Socio-Economic Status
NGO: Non-Governmental Organization
SD: Standard deviation
GMR: Geometric Mean Ratio
CI: Confidence Interval
BDT: Bangladeshi Taka
USD: United States Dollar
