# Supplementary material for: Cost of diabetes and hypertension care among patients in rural Bangladesh: a cross-sectional study
Source: BMC Public Health. 2026 Feb 4;26:806. doi: 10.1186/s12889-026-26456-8 (PMC12964931; doi:10.1186/s12889-026-26456-8)
Supplement: Supplementary file 1 — Supplementary Material 1. [file 12889_2026_26456_MOESM1_ESM.docx]

**Supplementary Figure 1: Population and health facility distribution of the study sites**


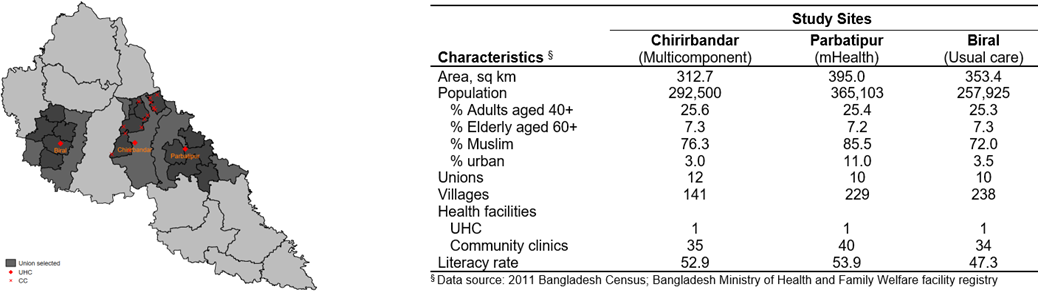


**Supplementary Figure 2: Analytic sample of the study**

Participants with complete information

(n= 6,849)

Participants with Hypertension (n= 2,690)

- Previously diagnosed with high BP by a health care provider (n= 1,114)
- Newly diagnosed with high BP by the study team (n=1,576)

Participants with Hypertension currently under treatment (taking medication)

(n= 635)

Participants with Diabetes (n= 967)

- Previously diagnosed with high blood sugar by a health care provider (n= 591)
- Newly diagnosed with high blood sugar by the study team (n=376)

Participants with Diabetes currently under treatment (taking medication)

(n= 335)

Total sample considered for the current study (n= 832)

**Supplementary Figure 3:** **Monthly time lost (in minutes) by patients and attendees for hypertension and diabetes care**


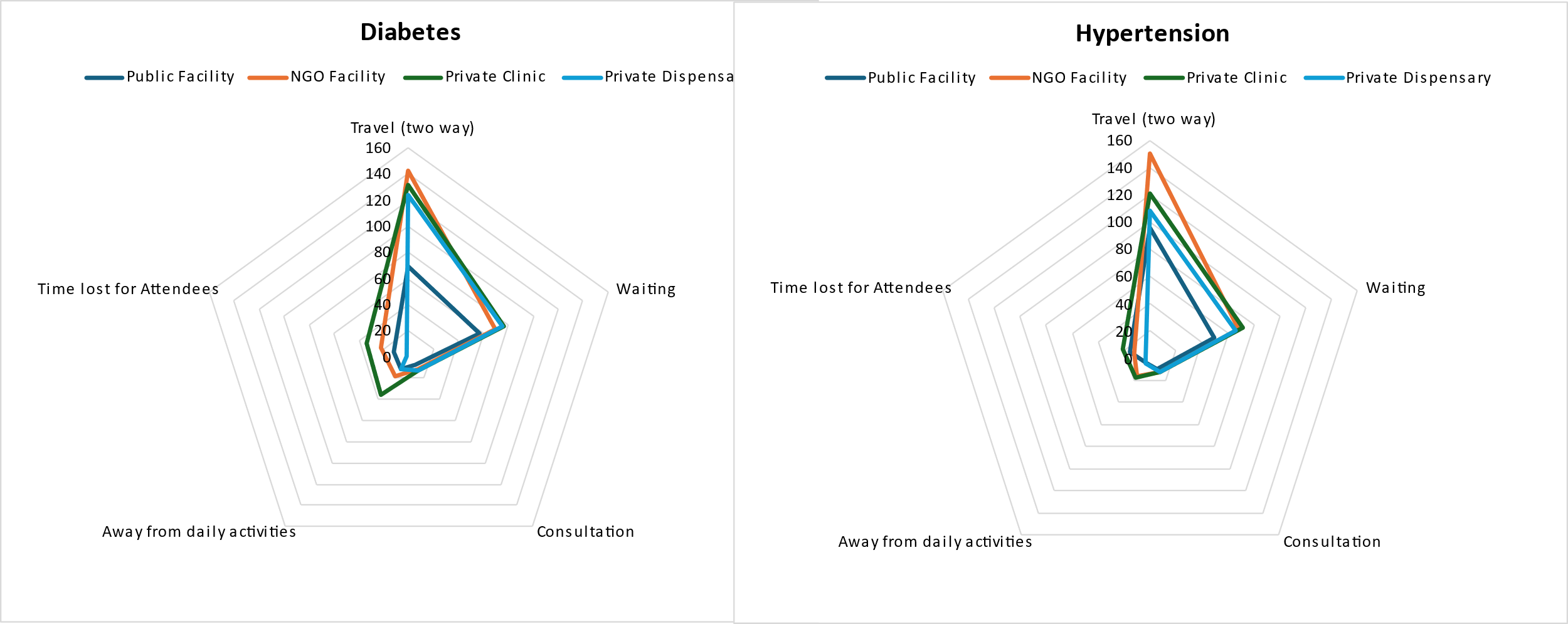


**Supplementary Table-1: Factors** **associated with the direct cost of seeking care for hypertension and diabetes (adjusted for zero-cost observation)**

| **Indicators** | **Hypertension** | | | **Diabetes** | |
| --- | --- | --- | --- | --- | --- |
|  | | **Model I** | **Model II** | **Model I** | **Model II** |
|  | | **GMR (95% CI)** | | | |
| **Age group (Ref: 40-49)** | |  |  |  |  |
| 50-59 | | 1.05 (0.75, 1.46) | 0.86 (0.52, 1.42) | 1.36 (0.97, 1.92) | 1.31 (0.81, 2.14) |
| 60+ | | 1.00 (0.70, 1.43) | 0.72 (0.39, 1.32) | 1.29 (0.84, 1.97) | 1.47 (0.86, 2.52) |
| **Sex (Ref: Male)** | |  |  |  |  |
| Female | | 0.94 (0.73, 1.21) | 0.71 (0.43, 1.16) | 1.09 (0.82, 1.46) | 0.85 (0.54, 1.33) |
| **Currently married (Ref: No)** | |  |  |  |  |
| Yes | | 0.97 (0.70, 1.36) | 1.26 (0.63, 2.51) | 1.37 (0.90, 2.09) | 2.19 (0.85, 5.65) |
| **Education (Ref: No formal schooling)** | |  |  |  |  |
| Primary | | 1.35 (1.07, 1.70) * | 1.41 (0.86, 2.31) | 1.48 (1.08, 2.02) * | 1.15 (0.76, 1.75) |
| Secondary or higher | | 1.37 (0.91, 2.05) | 1.20 (0.52, 2.75) | 1.76 (1.07, 2.92) * | 1.54 (0.80, 2.97) |
| **Employment status (Ref: Self-employed or homemaker)** | |  |  |  |  |
| Employed or retired | | 1.77 (1.20, 2.60) * | 0.84 (0.30, 2.35) | 1.36 (0.88, 2.11) | 0.63 (0.17, 2.42) |
| Unemployed | | 1.31 (0.89, 1.93) | 2.49 (1.41, 4.39) *** | 1.39 (0.84, 2.30) | 1.23 (0.60, 2.54) |
| **Wealth score (Ref: Low)** | |  |  |  |  |
| Medium | | 1.18 (0.92, 1.51) | 1.10 (0.68, 1.78) | 0.91 (0.56, 1.46) | 1.17 (0.48, 2.84) |
| High | | 1.43 (1.09, 1.87) * | 1.38 (0.78, 2.45) | 1.10 (0.82, 1.49) | 1.62 (0.76, 3.44) |
| **Having CVD (Ref: No)** | |  |  |  |  |
| Yes | | 1.78 (1.35, 2.35) *** | 1.68 (1.03, 2.74) ** | 1.74 (1.01, 3.01) * | 1.61 (0.62, 4.15) |
| **Having Chronic respiratory disease (Ref: No)** | |  |  |  |  |
| Yes | | 1.34 (0.98, 1.85) | 1.08 (0.60, 1.94) | 1.45 (0.87, 2.42) | 1.89 (0.77, 4.61) |
| **Having High cholesterol (Ref: No)** | |  |  |  |  |
| Yes | | 2.16 (1.49, 3.14) *** | 1.64 (0.77, 3.48) | 1.76 (1.11, 2.78) * | 1.07 (0.39, 2.93) |
| **Sources of care (Ref: Private Dispensary)** | |  |  |  |  |
| Public Facility | | 0.46 (0.33, 0.64) *** | 0.28 (0.15, 0.53) *** | 0.15 (0.10, 0.24) *** | 0.19 (0.09, 0.39) *** |
| NGO Facility | | 2.08 (1.37, 3.16) *** | 0.66 (0.27, 1.59) | 1.01 (0.72, 1.41) | 0.86 (0.50, 1.49) |
| Private Clinic | | 1.34 (1.06, 1.69) * | 0.78 (0.54, 1.13) | 1.07 (0.74, 1.52) | 1.20 (0.71, 2.04) |
| **Observations** | | 582 | 635 | 316 | 335 |
| **R-squared** | | 0.26 | 0.12 | 0.41 | 0.20 |

*Model I: Regression without adjustment to zero costs*

*Model II: Regression with adjustment to the zero costs (1 value added)*

*Note: p < 0.05*, p < 0.01**, p < 0.001****

**Supplementary Table-2: Factors associated with indirect cost of seeking care for hypertension and diabetes (adjusted for zero-cost observation)**

| **Indicators** | **Hypertension** | | | **Diabetes** | |
| --- | --- | --- | --- | --- | --- |
|  | | **Model I** | **Model II** | **Model I** | **Model II** |
|  | | **GMR (95% CI)** | | | |
| **Age group (Ref: 40-49)** | |  |  |  |  |
| 50-59 | | 1.22 (0.83, 1.79) | 0.79 (0.45, 1.38) | 1.13 (0.78, 1.64) | 0.80 (0.39, 1.65) |
| 60+ | | 1.03 (0.72, 1.47) | 0.93 (0.49, 1.74) | 0.99 (0.68, 1.43) | 0.60 (0.28, 1.32) |
| **Sex (Ref: Male)** | |  |  |  |  |
| Female | | 0.97 (0.74, 1.28) | 0.87 (0.50, 1.51) | 1.22 (0.87, 1.69) | 0.72 (0.38, 1.33) |
| **Currently married (Ref: No)** | |  |  |  |  |
| Yes | | 1.15 (0.80, 1.66) | 1.00 (0.60, 1.66) | 1.04 (0.68, 1.58) | 1.09 (0.43, 2.72) |
| **Education (Ref: No formal schooling)** | |  |  |  |  |
| Primary | | 1.41 (1.06, 1.88) * | 1.91 (1.13, 3.23) ** | 1.49 (1.02, 2.17) * | 1.82 (0.97, 3.42) * |
| Secondary or higher | | 1.41 (0.93, 2.12) | 1.58 (0.70, 3.54) | 2.19 (1.16, 4.13) * | 2.23 (0.84, 5.93) |
| **Employment status (Ref: Self-employed or homemaker)** | |  |  |  |  |
| Employed or retired | | 1.07 (0.72, 1.59) | 0.85 (0.43, 1.67) | 1.19 (0.68, 2.10) | 1.71 (0.62, 4.76) |
| Unemployed | | 1.11 (0.73, 1.68) | 1.72 (0.75, 3.97) | 0.97 (0.60, 1.57) | 0.96 (0.27, 3.43) |
| **Wealth score (Ref: Low)** | |  |  |  |  |
| Medium | | 0.89 (0.63, 1.25) | 0.79 (0.51, 1.22) | 0.87 (0.50, 1.52) | 0.92 (0.39, 2.16) |
| High | | 0.82 (0.64, 1.06) | 0.61 (0.35, 1.05) | 0.72 (0.49, 1.07) | 0.66 (0.26, 1.67) |
| **Having CVD (Ref: No)** | |  |  |  |  |
| Yes | | 1.27 (0.91, 1.76) | 1.02 (0.57, 1.84) | 1.76 (1.21, 2.54) *** | 2.63 (0.89, 7.81) * |
| **Having Chronic respiratory disease (Ref: No)** | |  |  |  |  |
| Yes | | 1.20 (0.78, 1.83) | 0.89 (0.48, 1.67) | 0.98 (0.63, 1.53) | 0.96 (0.37, 2.52) |
| **Having High cholesterol (Ref: No)** | |  |  |  |  |
| Yes | | 1.75 (1.20, 2.57) * | 1.07 (0.53, 2.16) | 1.46 (0.98, 2.19) | 0.96 (0.38, 2.42) |
| **Sources of care (Ref: Private Dispensary)** | |  |  |  |  |
| Public Facility | | 1.99 (1.40, 2.84) *** | 1.69 (0.86, 3.32) | 2.34 (1.52, 3.61) *** | 2.94 (1.13, 7.65) *** |
| NGO Facility | | 1.89 (1.27, 2.83) *** | 1.54 (0.73, 3.23) | 2.27 (1.43, 3.61) *** | 1.27 (0.63, 2.55) |
| Private Clinic | | 1.90 (1.38, 2.62) *** | 1.02 (0.62, 1.67) | 2.28 (1.55, 3.37) *** | 1.19 (0.52, 2.71) |
| **Observations** | | 414 | 635 | 236 | 335 |
| **R-squared** | | 0.15 | 0.03 | 0.19 | 0.06 |

*Model I: Regression without adjustment to zero costs*

*Model II: Regression with adjustment to the zero costs (1 value added)*

*Note: p < 0.05*, p < 0.01**, p < 0.001****

**Supplementary Table-3: Direct and indirect costs (monthly) of care among patients with hypertension, diabetes and both conditions [in BDT]**

| Cost Items | Hypertension only (n=497) | | Diabetes only (n=197) | | Both Hypertension and Diabetes (n=138) | |
| --- | --- | --- | --- | --- | --- | --- |
|  | Mean BDT (95% CI) | % of total cost | Mean BDT (95% CI) | % of total cost | Mean BDT (95% CI) | % of total cost |
| Direct Medical Cost Items |  |  |  |  |  |  |
| Registration | 4 (0.09 - 8.80) | 0.5% | 22 (8.61 - 35.37) | 1.3% | 24 (-0.62 - 48.97) | 0.9% |
| Consultation | 52 (36.25 - 66.77) | 5.4% | 47 (17.23 - 76.37) | 2.8% | 86 (40.93 - 131.54) | 3.3% |
| Diagnosis | 100 (55.03 - 145.79) | 10.6% | 230 (84.59 - 374.95) | 13.6% | 450 (98.83 - 802.11) | 17.4% |
| Medicine | 602 (518.47 - 686.03) | 63.4% | 990 (660.27 - 1319.54) | 58.5% | 1589 (1298.15 - 1880.21) | 61.2% |
| Total direct medical cost | **763 (646.54 -878.90)** | **80.3%** | **1324 (788.69 - 1859.32)** | **78.3%** | **2150 (1567.01 - 2733.10)** | **82.8%** |
| Direct Non-medical Cost Items |  |  |  |  |  |  |
| Travel | 44 (27.67 - 60.71) | 4.7% | 66 (38.82 - 92.90) | 3.9% | 89 (44.95 - 132.81) | 3.4% |
| Food cost | 44 (28.65 - 59.67) | 4.7% | 141 (71.05 - 210.83) | 8.3% | 165 (80.98 - 249.24) | 6.7% |
| Informal costs (gifts, tips) | 4 (-0.05 - 7.49) | 0.4% | 4 (-2.49 - 9.60) | 0.2% | 6 (-2.22 - 13.23) | 0.2% |
| Total direct non-medical cost | **92 (63.97 - 120.99)** | **9.7%** | **211 (114.54 - 307.19)** | **12.5%** | **260 (152.23 - 367.48)** | **10.0%** |
| Indirect Costs |  |  |  |  |  |  |
| Cost of lost time/productivity (patient) | 87 (74.24 - 99.39) | 9.1% | 145 (112.85 - 178.02) | 8.6% | 156 (114.76 - 196.46) | 6.0% |
| Cost of lost time/productivity (attendant) | 8 (3.63 - 12.09) | 0.8% | 11 (3.20 - 18.58) | 0.6% | 31 (12.45 - 48.94) | 1.2% |
| Total indirect cost | **95 (79.83 - 109.53)** | **10.0%** | **156 (120.26 - 192.38)** | **9.2%** | **186 (131.67 - 240.93)** | **7.2%** |
| Total cost | **950 (814.02 - 1085.73)** | **100.0%** | **1691 (1092.02 - 2290.35)** | **100.00%** | **2596 (1896.48 - 3295.95)** | **100.0%** |
| Total cost in USD | **8 (6.90 – 9.20)** |  | **14 (9.25 – 19.41)** |  | **22 (16.07 – 27.93)** |  |
